# Associations between Restrained Eating and the Size and Frequency of Overall Intake, Meal, Snack and Drink Occasions in the UK Adult National Diet and Nutrition Survey

**DOI:** 10.1371/journal.pone.0156320

**Published:** 2016-05-26

**Authors:** Ana Lorena Olea López, Laura Johnson

**Affiliations:** Centre for Exercise, Nutrition and Health Sciences, School for Policy Studies, University of Bristol, Bristol, United Kingdom; University of Tennessee Health Science Center, UNITED STATES

## Abstract

Obesity is a global public health priority. Restrained eating is related to obesity and total energy intake but associations with the eating patterns are unclear. We examined the associations of restrained eating with the size and frequency of intake occasions among 1213 British adult (19–64 y) participants in a cross-sectional analysis of the UK National Diet and Nutrition Survey 2000. The Dutch Eating Behaviour Questionnaire assessed restrained eating. Overall intake occasions were all energy consumed in a 60 min period. A food-based classification separated intake occasions into meals, snacks, or drinks from seven-day weighed food diaries. Average daily frequency and size (kcal) of overall intake, meal, snack and drink occasions were calculated and associations with restrained eating were modelled using multiple linear regression including under-reporting of energy intake, age, gender, BMI, emotional eating, external eating and physical activity as covariates. Restrained eating was very weakly positively correlated with overall intake (r = 0.08, p<0.05) and meal frequency (r = 0.10, p<0.05) but not snack or drink frequency (r = 0.02 and -0.02 respectively). Adjusted regressions showed a one-point change in restrained eating was associated with 0.07 (95% CI 0.03, 0.11) more meal occasions/day and 0.13 (95% CI 0.01, 0.25) extra overall intake occasions/day. Overall intake occasion size was weakly negatively correlated with restrained eating regardless of type (r = -0.16 to -0.20, all p<0.0001). Adjusted regressions showed each one-point increase in restrained eating was associated with lower-energy meals (-15 kcal 95% CI -5.9, -24.2) and drinks (-4 kcal 95%CI -0.1, -8), but not snacks or overall intake occasions. Among a national sample of UK adults, greater restrained eating was associated with smaller and slightly more frequent eating, suggesting that restrained eaters restrict their energy intake by reducing meal/drink size rather than skipping snacks.

## Introduction

Obesity is one of the most important public health problems worldwide and is the cause of at least 2.8 million global deaths [1]. In England only 35% of both men and women have a healthy weight [2], making obesity a public health priority.

Obesity is caused when energy intake exceeds energy expenditure on a long-term basis [3]. Eating in excess of needs is a complex process that can be influenced by cultural, social and psychological factors; therefore, theories have been developed to identify those factors that affect food intake, and thus body weight control. The theory of restrained eating describes the tendency of people to restrict food intake to lose weight or to prevent weight gain [4]. However, while some studies have observed inverse associations between higher restraint and obesity risk [5, 6], it has been suggested that chronic or excessive restriction may actually be a risk factor for weight gain and becoming overweight or obese [7–13]. A recent longitudinal study in a representative sample of Dutch adults found that in females, but not males, higher levels of restraint at baseline was associated with greater gains in BMI after 3 years follow-up [14]. However, in clinical studies of weight loss people randomised to an interventions focussed on relaxation of restraint tend to have poorer weight loss than people randomised to an intervention including restraint [15].

Consistent inverse correlations between restrained eating scores and total energy intake have been observed [16–18]. In addition, variation in diet quality, such as lower intakes of fat or energy dense foods that can reduce energy intake, has been observed among restrained eaters [19, 20]. Reducing meal size or frequency e.g. by avoiding snacks, are also hypothesised to reduce total energy intake. For example, snacks that add to existing food intake without a reduction in the size or frequency of subsequent eating will increase total energy intake [21]. Alternatively, snacking may reduce hunger throughout the day and result in smaller meals and lower total energy intake [22]. The extent to which restrained eaters regulate their energy intake by reducing the size or frequency of intake occasions has been studied just once before to our knowledge. De Castro, 1995, observed that cognitive restraint was associated, albeit weakly, with meal size but not meal frequency. The weak or null associations observed could be explained by the relatively small sample size (n = 358) or under-reporting of energy by restrained eaters caused by social desirability [23], which was not accounted for in the analysis [24]. A further limitation was that all meals were considered together whereas meals, snacks and drinks may have different effects on the regulation of energy intake [21, 25, 26]. Understanding how restrained eaters achieve a lower energy intake could highlight promising targets for successful prevention of weight gain. Therefore, we analysed associations of restrained eating with the size and frequency of overall intake occasions, accounting for under-reporting, in a representative sample of UK adults. We also used a food-based classification to identify meal, snack and drink intake occasions. We hypothesised that restrained eaters would have smaller and less frequent intake occasions, specifically by having fewer snack intake occasions.

## Materials and Methods

### Sample

The National Diet and Nutrition Survey (NDNS) 2000 program cross-sectionally surveyed a nationally representative sample of adults (aged 19–64 years) selected using a multistage random probability design living in private households across the UK about their diet, nutritional status and nutrient intake. Further details about the sample design and data collection can be found elsewhere [27]. In brief, fieldwork was conducted over a 12-month period in 2000/2001 by trained interviewers asking participants to complete a 7-day weighed dietary record of food intake both in and out of the home. A set of accurately calibrated weighing scales were provided along with instructions on how to weigh all food, drinks and leftovers and record details on brand names and cooking methods. Ethical approval for the NDNS was obtained from the Multi-centre Research Ethics Committee and National Health Service Local Research Ethics Committee covering each of the 152 postcodes areas in the sample. For the present analysis, the dataset is publicly Available the National Data archive after project approval [28]. Local ethical approval for the current analysis was given by the University of Bristol Centre for Exercise, Nutrition and Health Sciences Research Ethics Committee (approval number: EAN 026–13).

### Size and frequency of meal, snack and drink intake

An intake occasion was considered as everything consumed within each 60 minute period starting from the first reported time in an individual record day. A food based definition was used to identify intake occasions as meals, snacks or drink only [29]. All NDNS food groups were allocated either to a meal list, snack list or drink list (S1 File), based on frequently consumed foods during meals and snacks [29–31]. For this analysis supplements were excluded. Intake occasions were labelled as meal, snack and drink based on the following criteria:

Meal: If all items in an occasion were from the meal list. Or if an occasion contained more than one item and at least one item consumed was from the meal-list combined with items from either the snack or drink list (except where only 2 items are reported and one is a meal food and one is a snack e.g. bread and butter, which was classified as a snack).

Snack: If all items in an occasion were from the snack list. Or if an occasion contained two items, one from the meal-list and one from the snack-list e.g. bread and butter.

Drink: If all items in an occasion were from the drink list. If an intake occasion contained 2 items and one was a snack (sugar) and one was a drink item (tea/coffee).

The average size of each intake occasion was calculated by summing the energy (kcal) in all food/drink items consumed and averaged per person across 7 days for overall occasions and separately for meals, snacks, and drink occasions. The total number of overall intake occasions was counted each day and averaged over 7 days for each participant to represent average daily overall intake frequency. The frequency of occasions defined as meals, snacks, and drinks were also counted and then averaged separately.

### Restrained eating

After diet diary completion participants were asked to complete the Dutch Eating Behaviour Questionnaire (DEBQ) [32]. The DEBQ consists of 33 items assessing emotional (13 items), external (10 items) and restrained eating (10 items) on a 5-point Likert scale (1 = never, 2 = seldom, 3 = sometimes, 4 = often, 5 = very often). Restrained eating score was calculated as the mean of the 10 responses of the restrained eating subscale. Scores were categorised as high (>3) and low (≤3) for some analyses [23]. High internal reliability for restrained eating was found in this sample, Cronbach’s α coefficients of the scales were 0.91.

### Covariates

Physical activity diaries were completed during the same 7-days of the dietary record. Height (cm) and weight (kg) were measured twice in light clothing with shoes and socks removed using a Leicester Height measure and Soehnle Quantratonic Scales (Models 7300 and 7306, Soehnle-Waagen GmbH & Co). Body mass index (BMI) was calculated (weight(kg)/height(m)2) and subjects were defined as healthy (BMI<25), overweight (BMI 25–29.9) or obese (BMI> = 30). Ethnicity was self-defined in the questionnaire and social class was based on self-reported occupation and defined as manual or non-manual using the National Statistics Socioeconomic Classification [33]. Estimated energy requirements were calculated for each participant based on sex, age, weight, height and physical activity level (PAL) using standard equations [34]. The equations require PAL to be defined for each participant as one of four categories (sedentary, low active, active or very active). Physical activity diaries, where participants reported all activities and their duration for 7 days, were used to calculate metabolic equivalent-hours per week (METs) [27]. METs were converted to PAL, which was used to allocate participants to one of the 4 IOM categories. Reported total energy intake (TEI) was divided by estimated energy requirements (EER) (TEI/EER), which under the assumption of energy balance should equal 1. To account for known sources of random error in the estimation of energy intake and expenditure, coefficients of variation were used to calculate confidence limits of agreement for EI/EER. Any values of EI/EER below 0.71 were defined as under-reporting and values above 1.29 were defined as over-reporting [35].

### Statistical analyses

Data are presented as means and standard deviations for scale variables or frequencies and percentages for categorical variables. Differences in scale variables between 2 groups were examined using independent t-tests. Pearson’s correlation coefficients measured the simple linear relationship between two scale variables. To explore independent relationships multiple linear regressions were performed using restrained eating score as the independent variable and with the size or frequency of overall intake occasions or meals or snack or drinks as the outcome variable in their own models. All analyses were controlled for age, gender, BMI, physical activity, emotional eating scores and external eating scores (Model 1). Under-reporting category was added in the most adjusted models (Model 2). Unstandardized betas (β) from these models are presented. Interactions between restrained eating score and gender for all eating patterns were tested by including a product term in regression models. The criterion for significance was p<0.05. As overall intake, meal, snack and drink frequencies may also be considered discrete ordinal rather than as scale variables, sensitivity analyses were run using ordinal logistic regression models with covariates adjusted for as previously described for models 1 and 2. Scale average frequency variables were rounded to the nearest whole occasion and categories with small numbers of participants were collapsed to create the following ordinal variables: overall intake frequency = <4; 5; 6; 7; 8; 9; 10; 11+ occasions per day; meal frequency 1; 2; 3; 4+ meals per day; snack frequency 1; 2; 3; 4+ snacks per day; and drink frequency 1; 2; 3; 4; 5+ drinks per day. Analyses were completed in SPSS version 23 (SPSS, Inc., Chicago, IL, USA). Research has been reported following STROBE guidelines (checklist in S1 Table).

## Results

From the 1724 cases who completed the dietary record, those with missing data on DEBQ (n = 96), anthropometric data (n = 73) and physical activity diaries (n = 51) were excluded. Dieters (those who confirmed they were dieting to lose weight at that moment, n = 292) were excluded not only to minimize under-reporting bias [23] but also to avoid a limitation on identifying eating patterns, as their current intake may not have represented habitual intake. The final sample size included for analysis was n = 1213. Table 1 presents the relevant participants’ characteristics separately by sex and restrained eating score. The adult respondents of the NDNS were 19–64 years old and 94% of the sample were White British. More than half of the population were either obese (18.2%) or overweight (36.7%). People with high restrained eating scores were more likely to be male, older and have non-manual occupations (Table 1). EER for women was 2293 kcal (SD = 233) and the average energy intake was 1687 (SD = 426), therefore reported energy intakes represented around 74% of the EER. In the case of men, the mean EER was 3101 kcal (SD = 355), the average TEI was 2367 kcal (SD = 591) and represented 77% of the EER. Under-reporting of energy intake was greater in high restrained males compared with low restrained males, but did not differ by levels for restrained eating in women (Table 1).

**Table 1:** Descriptive statistics for participants included in the analysis.

	Male (n = 586)			Female (n = 627)		
	Restrained Eating			Restrained Eating		
	Low	High	p	Low	High	p
N (%) ^a^	514 (92%)	72 (8%)		487 (78%)	140 (22%)	<0.0001^b^
Age (years) ^c^	41 (12)	45 (12)	0.012	41 (12)	45 (12) ^b^	0.001
BMI (kg/m^2^) ^c^	26.6 (4.4)	28.2 (3.9)	0.003	25.6 (5.8)	26.2 (5.1)	0.216
Social class (% manual) ^a^	217 (43%)	23 (32%)	0.048	201 (43%)	45 (32%)	0.017
Restrained eating score ^c^	1.8 (0.6)	3.5 (0.4)	<0.0001	2.0 (0.7)	3.6 (0.5)	<0.0001
Emotional eating score ^c^	1.6 (0.6)	1.8 (0.6)	0.001	1.9 (0.7)	2.2 (0.7)	<0.0001
External eating score ^c^	2.5 (0.7)	2.7 (0.5)	0.091	2.6 (0.6)	2.7 (0.6)	0.049
Physical activity score (METs) ^c^	45.9 (9.8)	46.3 (11.1)	0.721	42.2 (3.9)	42.2 (3.7)	0.813
TEI (kcal) ^c^	2386 (602)	2231(488)	0.043	1686 (441)	1690 (368)	0.916
TEI/EER (%) ^c^	78 (20)	71 (16)	0.005	74 (20)	74 (16)	0.757
Overall intake frequency (per day) ^c^	7.0 (1.9)	6.8 (1.8)	0.370	6.8 (1.8)	7.3 (1.7)	0.002
Meal frequency (per day) ^c^	2.6 (0.7)	2.7 (0.6)	0.301	2.5 (0.6)	2.6 (0.6)	0.014
Snacks frequency (per day) ^c^	2.1 (1.1)	2.1 (1.0)	0.898	2.1 (0.9)	2.1 (0.9)	0.458
Drinks frequency(per day) ^c^	3.0 (1.4)	2.7 (1.3)	0.104	2.9 (1.4)	3.1 (1.3)	0.105
Overall intake occasion size (kcal) ^c^	356 (110)	347 (110)	0.477	258 (82)	238 (63)	0.009
Meal size (kcal) ^c^	703 (168)	652 (124)	0.013	520 (126)	496 (99)	0.043
Snacks size (kcal) ^c^	245 (163)	221 (166)	0.283	219 (180)	193(121)	0.057
Drinks size (kcal) ^c^	97 (66)	92(66)	0.637	62 (64)	50 (44)	0.005

^a^ Values are frequency (%). Differences between low and high restrained eaters tested within sex using χ^2^ test.

^b^ Association between high and low restrained eating and sex was tested using a χ^2^ test p<0.0001.

^c^ Values are Mean SD. Differences in means between low and high restrained eaters tested within each sex using independent samples t tests.

**Fig 1:**
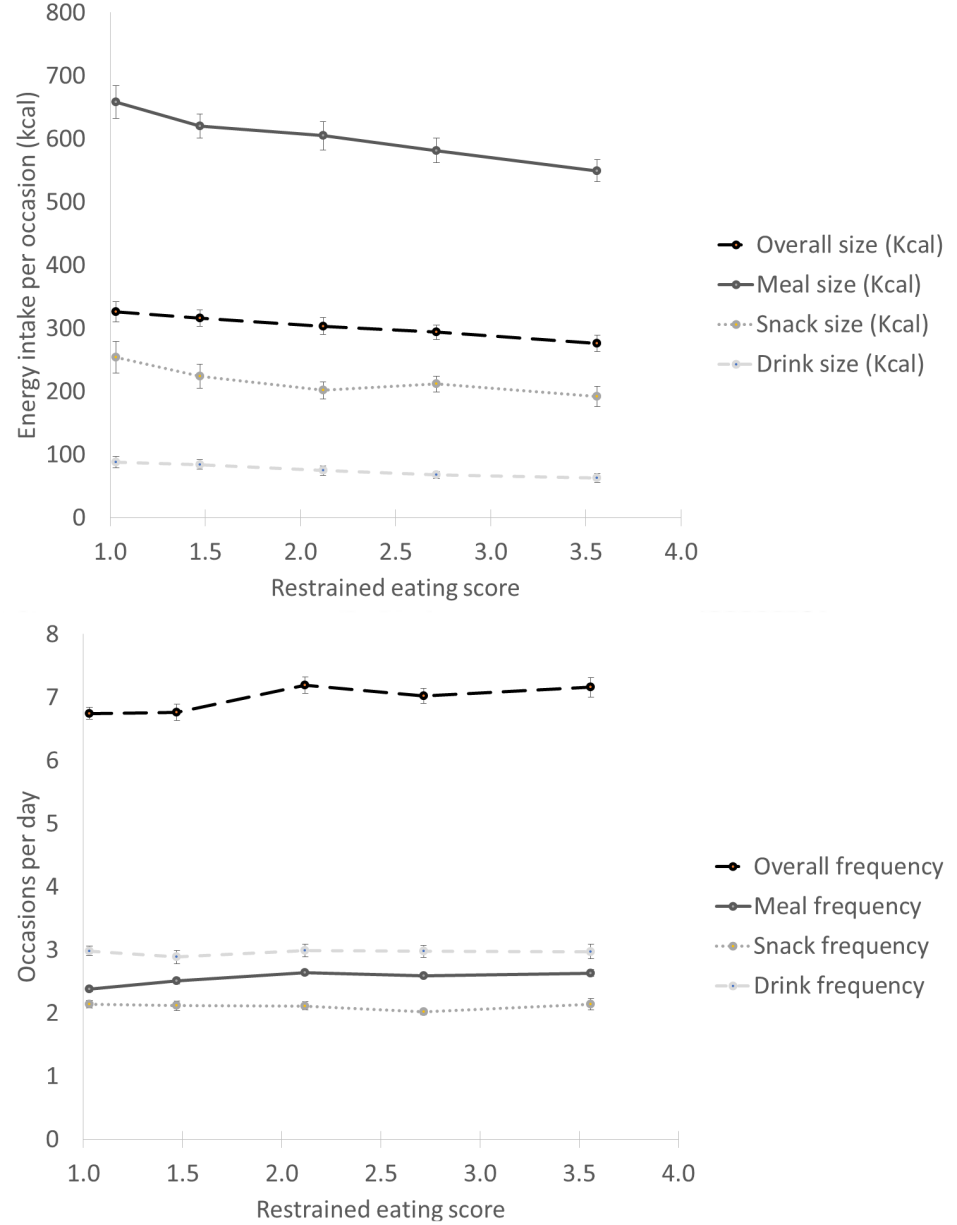
Overall intake, meal, snack, and drink size and frequency by quintiles of restrained eating score.

Weak but positive correlations were found for restrained eating score with overall intake and meal frequency (Table 2). There was no correlation of restrained eating with snack or drink frequency. In contrast stronger (but still weak) correlations were observed for restrained eating with overall intake, meal, snack and drink size, with the largest correlation observed for meal size (r = -0.20, p<0.0001). The linear trends for overall intake, meal, snack, and drink size and frequency by quintiles of restrained eating score is displayed in Fig 1 and supports an inverse association of restrained eating with overall intake occasion size, driven largely by differences in meal size (difference between quintile 1 and 5 was 109 kcal for meal sizes).

**Table 2:** Pearson correlations between restrained eating score and eating pattern variables.

Variables	Restrained eating	Overall intake frequency	Meal frequency	Snack frequency	Drink frequency	Overall intake size	Meal size	Snack size
Overall intake frequency	0.08^b^							
Meal frequency	0.10^a^	0.24^a^	-	-	-	-	-	-
Snack frequency	-0.02	0.46^a^	-0.12^a^	-	-	-	-	-
Drink frequency	0.02	0.71^a^	-0.08^b^	-0.08^b^	-	-	-	-
Overall intake size	-0.16^a^	-0.49^a^	-0.16^a^	-0.24^a^	-0.42^a^	-	-	-
Meal size	-0.20^a^	-0.01	-0.22^a^	-0.06^b^	-0.08^b^	0.63^a^	-	-
Snack size	-0.12^a^	-0.21^a^	-0.09^a^	-0.27^a^	-0.01	0.45^a^	0.30^a^	-
Drink size	-0.16^a^	-0.16^a^	-0.10^a^	0.05	-0.09^a^	0.43^a^	0.30^a^	0.11^a^

^a^ Statistically significant (p<0.0001).

^b^ Statistically significant (p<0.05).

In multiple linear regression models adjusted for age, gender, BMI, physical activity, emotional eating scores and external eating scores very small but significant associations were observed for the frequency of meal occasions only (Table 3). After adjustment for under-reporting (Model 2, table 3) the association estimate between restrained eating and overall intake frequency almost doubled (0.07 vs 0.13) and became statistically significant. The association with meal frequency was also slightly stronger (0.05 vs. 0.07). A one unit increase in restrained eating score was associated with a 0.13 and 0.07 increase in overall intake and meal eating occasions respectively.

**Table 3:** Regression models for the association of the frequency and size of intake occasions with restrained eating score.

	Model 1^a^		Model 2^b^	
	B	*(95% CI)*	B	*(95% CI)*
Overall intake frequency (per day)	0.07	(-0.06, 0.19)	0.13	(0.01, 0.25)
Meal frequency (per day)	0.05	(0.01, 0.09)	0.07	(0.03, 0.11)
Snack frequency (per day)	-0.06	(-0.13, 0.01)	-0.05	(-0.12, 0.02)
Drink frequency (per day)	0.06	(-0.04, 0.16)	0.07	(-0.03, 0.17)
Overall intake occasion size (kcal)	-8.6	(-2.2, -15.0)	-5.2	(-11.4, 0.9)
Meal size (kcal)	-20.3	(-29.9, -10.6)	-15	(-24.2, -5.9)
Snack size (kcal)	-10.2	(-19.7, -0.7)	-8.8	(-18.3, 0.7)
Drink size (kcal)	-4.4	(-8.1, -0.7)	-3.8	(-7.5, -0.1)

^a^ Model 1: The association of restrained eating controlling for age, gender, BMI, emotional eating scores, external eating scores and physical activity levels.

^b^ Model 2: The association of restrained eating controlling for age, gender, BMI, emotional eating scores, external eating scores, physical activity levels and underreporting category.

In multiple linear regression models adjusted for age, gender, BMI, physical activity, emotional eating scores and external eating scores significant associations were observed for the size of intake occasions regardless of whether overall intake, meal, snack or drink (Table 3). After adjustment for under-reporting these association estimates were slightly attenuated but remained significant for meal and drink size. An increase in one unit of restrained eating was associated with 15 kcal and 4 kcal reduction in the average size of meal and drink occasions, respectively. No interaction tests for differences in associations by gender were statistically significant (all p>0.09).

Sensitivity analyses using ordinal logistic regression models found estimates of a broadly similar direction and statistical significance. After adjustment for model 2 covariates each one unit increase in restrained eating score was associated with being in a higher overall intake, meal or drink frequency category with an ordered odds of 1.16 (95% CI 1.02, 1.31); 1.36 (95% CI 1.02, 1.31) and 1.14 (95% CI 1.00, 1.29) respectively. There was no evidence of association between restrained eating and snack frequency (ordered odds 0.96 (95% CI 0.84, 1.09)).

## Discussion

In a large nationally representative sample of UK adults we found higher restrained eating scores were associated with smaller overall intake occasions and a slightly higher overall intake frequency and meal frequency, indicating a shift towards a smaller but more frequent pattern of consumption in restrained eaters. However, the correlation between restrained eating and overall intake occasion size was double the size of the correlation with overall intake frequency, suggesting that restrained eaters restrict their energy intake most often by reducing portion sizes rather than skipping meals or snacks. Regression models adjusted for external eating, emotional eating, BMI, sex, age, physical activity, and underreporting found that associations between restrained eating score and the size of intake occasions was specific to meals and drinks, but not snacks or overall intake occasions combined. Similarly, while meal eating occasion frequency increased slightly with greater restrained eating, the frequency of snacks was not associated with restrained eating.

It was hypothesized that restrained eating would be inversely related with overall intake frequency as a result of skipping meals or snacks. In contrast, we found very weak positive associations. While statistically significant in regression models, a 1 point change in restrained eating was associated with a change of only 0.13 overall intake occasions, which is equivalent to approximately one extra occasion per week, a difference too small to be meaningful in real life. Our findings are broadly in agreement with de Castro (1995) who found that meal frequencies did not differ by level of restrained eating assessed with the Three Factor Eating Questionnaire [24]. The absence of associations in the de Castro paper could be explained by a smaller sample size, which was a quarter of the size of the sample in the current analysis.

Murakami and Livingstone (2014) have also examined eating frequency in the same study (NDNS), but in a slightly larger sample (1487 vs. 1213) and using a different definition of intake occasions. Murakami & Livingstone (2014) defined intake occasions as any food or drink consumed in the same 15 min period, whereas we defined intake occasions as everything consumed within each 60 minute period. We also used a food based approach to separate intake occasions into specific types namely, meals, snacks and drinks. Therefore our definition inherently limits people to a maximum of 24 overall intake occasions compared with up to 96 possible intake occasions [36]. Despite these differences, the average overall intake frequency observed in our analysis was similar at around 7 intake occasions a day [36]. Also similar to Murakami and Livingstone 2014, we observed a stronger association between overall intake frequency and restrained eating after adjustment for under-reporting, suggesting this is an important feature of observational studies of eating frequency to ensure robust results.

Restrained eating may reflect eating less than desired, which may be higher than energy intakes required by the body for weight maintenance. However, in our analysis we observed reported total energy intakes were even lower when compared with estimated energy requirements for high vs. low restrained eaters. Under-estimation of energy intake is a well-documented phenomenon that occurs with all dietary assessment tools as a result of both under-reporting of food that was truly eaten and actual under-eating of food during the assessment period compared to habitual energy intake [37]. Restrained eating has been associated with greater underestimation of energy intakes both in a previous analysis of the National Diet and Nutrition Survey[23] and in another study where energy expenditures were measured directly with doubly labelled water[38], which suggests the association is not driven by inherent problems with the estimating energy requirements. Studies of the construct validity of restrained eating score found that the DEBQ score was associated with successful caloric restriction in everyday life [15], which may mean that the lower energy intakes observed could be expected based on the higher levels of restraint,

Overall intake occasion size was associated with restrained eating score, an association that was driven largely by an increase in meal sizes. Meal size and restrained eating were similarly correlated in De Castro (1995) although he reported a slightly stronger correlation (r = -0.31). We have extended the work of de Castro by separating overall intake occasions into meals, snacks and drinks based on the food and drinks consumed within each occasion. This has allowed the association of restrained eating with snacks and drinks to be studied for the first time and has revealed limited evidence that snacks play an important role in explaining differences in eating patterns by restrained eating score.

According to our results from the most adjusted model restrained eaters appeared to restrict the amount of calories consumed during meals and drinks more consistently than in snacks. Equally the association of restrained eating with eating frequency appeared to be driven by a lower meal frequency, whereas the frequency of snacks varied little across level of restrained eating. The specific effect on drinks could be explained by restrained eaters switching from sugar-sweetened to artificially sweetened drinks, as has been observed in a Swedish sample [39]. The association with smaller size and frequency of meals may be associated with the combined effect of the impulsive vs. reflective system response to food intake, where successful snack restriction is disrupted when cognitive resources are depleted by impulsive behaviour driven by implicit liking for snack foods [40]. Furthermore, previous research into the types of foods typically consumed by restrained eaters highlights lower intakes of fats, oils, red meat, pizza, french fries and full-fat dairy [19], which are foods most likely to be combined within meals rather than eaten alone as a snack. Therefore the combined effect of restrained eating across all foods maybe more likely to affect meals than snacks.

Alternatively, the absence of associations between restrained eating and snack size and frequency may be related to the definition of snack that we used. Our definition of snack foods included both high and low quality snack foods i.e. biscuits and cakes as well as fresh fruit and raw vegetables. If restrained eaters chose to substitute a low quality snack for a high quality snack rather than skipping snacks all together then snack frequency would be unchanged. However, if this were the case then one would expected the amount of calories in a snack to be reduced when a higher quality snack food was eaten as they are typically less energy dense. While we initially observed an association between restrained eating and snack size it was not robust to adjustment for under-reporting of energy intake. Further research should specifically explore the combined associations of eating frequency and diet quality with restrained eating.

Differences in meal and drink size were not reflected in differences in overall intake occasion size in our most adjusted model. In our initial model both lower overall intake occasion and snack size were associated with higher restrained eating but these associations were not robust to adjustment for under-reporting of energy intake. Associations of restrained eating with overall intake occasion size represent the combination of meal, drink and snack size associations. Drinks were the most frequently consumed intake occasion but had relatively small associations in terms of size. Snacks were almost as frequent as meals but had an effect estimate of half the size of the effect of meals, which was attenuated further after adjustment for under-reporting. Therefore associations between restrained eating and meal size are muted when combined with drink and snack size such that for overall intake occasion size there is no evidence of association. This finding potentially emphasises the importance of treating eating occasions separately as the behavioural determinants of meals, snacks and drinks as may differ.

Average restrained eating scores among men and women in our population is difficult to compare with other cohorts as few other nationally representative surveys have reported restrained eating measured by the DEBQ. One large sample of Dutch adult males and females recruited from a panel survey reported average levels of restrained eating of 2.5–3.0, in line with our results [41]. In our analysis, women had higher restrained eating scores than men, which confirms existing evidence that has repeatedly shown women have higher restraint than men [11, 17, 18]. In terms of total energy intake, our results suggest that men with high scores for restrained eating had lower energy intakes compared with men with low restrained eating scores, whereas among women energy intake was no different by level of restrained eating. Tepper et al. (1996) reported that energy intake for women did not vary between restrained and unrestrained eaters however, restrained men consumed 398 kcal/day less than unrestrained men. It is possible, based on [19], that men are more likely to restrict effectively compared with women and may explain why dietary restraint is positively associated with an increase in BMI in women but not in men [14]. However, while there was a suggestion that overall intake frequency was higher and drink size smaller in restrained eating females compared with males, there was no evidence of an interaction by gender for any associations observed, which is in line with de Castro (1995). On the contrary, Tepper et al., (1996) found the association between restrained eating and snack foods (popcorn, chips, french fries and soda) differed significantly by gender. The difference with this study is that we classified intake occasions as snack, meal or drink based on a much wider range of foods. By not looking at specific food items gender preferences may have been missed. If there are gender differences in the association between the size and frequency of consumption and restrained eating then they are likely to be subtle as they were too small to be detected in the current sample of more than 1000 adults with precise measures based on weighed food intakes.

We observed that higher restrained eating was associated with smaller drink sizes, which could mean that restrained eaters have drinks that are sugar-free or low-calorie (e.g. water, low calorie soft drinks), or they may refrain adding sugar to tea and coffee. De Castro (1995) found a positive association between restrained eating and diet soda intake, which provides support to this hypothesis. In order to better understand the combined effect of eating patterns and food choice on restrained eating, future research could use more detailed meal coding to integrate information on the combinations of types of food or drink consumed within intake occasions, as well as their size and frequency [42].

The main limitation of this study is the potential for self-report bias related to physical activity and dietary diaries. We adjusted for physical activity categories to reduce the effect of overestimation based on information from a doubly labelled water sub-study in NDNS [35]. We also considered under-reporting of energy intake by adjusting for it in the multiple regressions, which should increase confidence that the eating patterns identified in this study are closer to usual intakes in the UK adult population. We aimed to explore how restrained eating affected eating patterns and by controlling for underreporting we might have missed periods of actual restriction [23]. A key strength of our analysis is the use of a large nationally representative sample of adults with 7 days of weighed dietary records. However, as data was collected in 2000–2001 it is not contemporary and associations may differ now. De Castro (1995) reported similar associations using data collected in the early 1990s giving support that associations between restrained eating and eating patterns are unlikely to be changing over time. Finally, as a cross-sectional study, causality cannot be inferred and thus longitudinal studies are required to understand the importance of these associations.

## Conclusions

In this cross-sectional study in British adults, we showed restrained eating was associated with smaller meal and drink sizes and, albeit weakly, more frequent overall intake occasions and meals. People with high restrained eating therefore appear to have an eating pattern characterised primarily by smaller and slightly more frequent intake. Understanding the techniques used to restrict energy intake in a free-living context can inform public health professionals on feasible ways of promoting eating patterns that are more likely to reduce overweight and obesity.

## Supporting Information

S1 File List of NDNS food groups used in the food based classification of intake occasions as meals, snacks or drink only. (DOCX)

S1 Table STROBE checklist. (DOC)
