# Supplementary material for: Associations between Restrained Eating and the Size and Frequency of Overall Intake, Meal, Snack and Drink Occasions in the UK Adult National Diet and Nutrition Survey
Source: PLoS One. 2016 May 26;11(5):e0156320. doi: 10.1371/journal.pone.0156320 (PMC4882017; doi:10.1371/journal.pone.0156320)
Supplement: S1 File — (DOCX) [file pone.0156320.s001.docx]

List of NDNS food groups used in the food based classification of intake occasions as meals, snacks or drink only

| **Meal food** | **Snack food** | **Drink** |
| --- | --- | --- |
| Bacon & ham | Apples pears | Alco-pops |
| Baked beans | Bananas | Beers |
| Beef, veal | Biscuits | Other beverage |
| Burgers kebab | Block marge | Bottled water |
| Carrot | Buns cakes pastries | Cider perry |
| Chicken & turkey | Chocolate con | Coffee |
| Coated chicken | Cottage cheese | Common toddler drinks |
| Common toddler food | Cream | Fortified wine |
| Cooked tomatoes | Fromage frais | Fruit juice |
| Egg dishes | Fruit in juice | Herbal tea |
| Eggs | Fruit in syrup | Liqueurs |
| Fried white fish | Fruit pies | Low alc beers |
| Green beans | Ice cream | Low alc cider perry |
| Lamb | Low fat spread | Low alc wine |
| Leafy green | Milk puds | Other milk |
| Liver | Nuts & seeds | Semi-skimmed |
| Meat pies etc | Oils&fats not pufa | Skimmed milk |
| Non fried potato products | Oranges | Soft drink fizzy diet |
| Oily fish | Other dairy dessert | Soft drink fizzy non-diet |
| Other white fish | Other cheese | Soft drink squash diet |
| Other breakfast cereals | Other fruit | Soft drink squash non-diet |
| Other bread | Other puds | Soft drink still diet |
| Other cereal | Other salad | Soft drink still non-diet |
| Other fried pots | Other sugars | Spirits |
| Other meat | Preserves | Tap water |
| Other potato dishes | PUFA low fat spread | Tea |
| Other veg | PUFA marge | Whole milk |
| Pasta | PUFA oils | Wine |
| Peas | PUFA reduced fat spread |  |
| Pizza | Raw carrots |  |
| Pork | Raw tomatoes |  |
| Potato chips | Reduced fat spread |  |
| Rice | Savoury sauces |  |
| Sausages | Savoury snacks |  |
| Shellfish | Soft marge not PUFA |  |
| Softgrain bread | Sponge puddings |  |
| Soups | Sugar |  |
| Vegetable dishes | Sugar confectionery |  |
| High fibre breakfast cereal | Sweeteners |  |
| White bread | Yogurt |  |
| Wholemeal bread | Butter |  |
