# Supplementary material for: Associations between Restrained Eating and the Size and Frequency of Overall Intake, Meal, Snack and Drink Occasions in the UK Adult National Diet and Nutrition Survey
Source: PLoS One. 2016 May 26;11(5):e0156320. doi: 10.1371/journal.pone.0156320 (PMC4882017; doi:10.1371/journal.pone.0156320)
Supplement: S1 Table — (DOC) [file pone.0156320.s002.doc]

STROBE Statement—Checklist of items that should be included in reports of ***cross-sectional studies***

|  | Item No | Recommendation | Location |
| --- | --- | --- | --- |
| **Title and abstract** | 1 | (*a*) Indicate the study’s design with a commonly used term in the title or the abstract | Line 34 |
|  |  | (*b*) Provide in the abstract an informative and balanced summary of what was done and what was found | Abstract |
| Introduction | | |  |
| Background/rationale | 2 | Explain the scientific background and rationale for the investigation being reported | Pg 3-4 |
| Objectives | 3 | State specific objectives, including any prespecified hypotheses | Pg 4 line 93-7 |
| Methods | | |  |
| Study design | 4 | Present key elements of study design early in the paper | Pg 4 |
| Setting | 5 | Describe the setting, locations, and relevant dates, including periods of recruitment, exposure, follow-up, and data collection | Pg 4 line 106-8 |
| Participants | 6 | (*a*) Give the eligibility criteria, and the sources and methods of selection of participants | Pg 4 line 119-24 |
| Variables | 7 | Clearly define all outcomes, exposures, predictors, potential confounders, and effect modifiers. Give diagnostic criteria, if applicable | Pg 5-6 |
| Data sources/ measurement | 8* | For each variable of interest, give sources of data and details of methods of assessment (measurement). Describe comparability of assessment methods if there is more than one group | Pg 5-6 |
| Bias | 9 | Describe any efforts to address potential sources of bias | Pg 6 line 167-78 |
| Study size | 10 | Explain how the study size was arrived at | Pg 4 line 119-24 |
| Quantitative variables | 11 | Explain how quantitative variables were handled in the analyses. If applicable, describe which groupings were chosen and why | Pg 5-6 |
| Statistical methods | 12 | (*a*) Describe all statistical methods, including those used to control for confounding | Pg 6 line 187-9 |
|  |  | (*b*) Describe any methods used to examine subgroups and interactions | Pg 6 line 190-1 |
|  |  | (*c*) Explain how missing data were addressed | Page 4 line 120-1 |
|  |  | (*d*) If applicable, describe analytical methods taking account of sampling strategy | NA |
|  |  | (*e*) Describe any sensitivity analyses | Pg 6 line 189-90 |
| Results | | |  |
| Participants | 13* | (a) Report numbers of individuals at each stage of study—eg numbers potentially eligible, examined for eligibility, confirmed eligible, included in the study, completing follow-up, and analysed | Pg 4 line 119-24 |
|  |  | (b) Give reasons for non-participation at each stage | Pg 4 line 119-24 |
|  |  | (c) Consider use of a flow diagram | Data shown Pg 4 line 120-22 |
| Descriptive data | 14* | (a) Give characteristics of study participants (eg demographic, clinical, social) and information on exposures and potential confounders | Table 1 |
|  |  | (b) Indicate number of participants with missing data for each variable of interest | Pg 4 line 120-22 |
| Outcome data | 15* | Report numbers of outcome events or summary measures | Table 1 |
| Main results | 16 | (*a*) Give unadjusted estimates and, if applicable, confounder-adjusted estimates and their precision (eg, 95% confidence interval). Make clear which confounders were adjusted for and why they were included | Table 1-3 |
|  |  | (*b*) Report category boundaries when continuous variables were categorized | Page 6 line 163-4 |
|  |  | (*c*) If relevant, consider translating estimates of relative risk into absolute risk for a meaningful time period | NA |
| Other analyses | 17 | Report other analyses done—eg analyses of subgroups and interactions, and sensitivity analyses | Pg 10 Line 241-242 |
| Discussion | | |  |
| Key results | 18 | Summarise key results with reference to study objectives | Page 11 line 245-55 |
| Limitations | 19 | Discuss limitations of the study, taking into account sources of potential bias or imprecision. Discuss both direction and magnitude of any potential bias | Pg 11-13 |
| Interpretation | 20 | Give a cautious overall interpretation of results considering objectives, limitations, multiplicity of analyses, results from similar studies, and other relevant evidence | Pg 13 line 26-30 |
| Generalisability | 21 | Discuss the generalisability (external validity) of the study results | Pg 13 line 24-26 |
| Other information | | |  |
| Funding | 22 | Give the source of funding and the role of the funders for the present study and, if applicable, for the original study on which the present article is based | Pg 14 line 341-52 |

*Give information separately for exposed and unexposed groups.

**Note:** An Explanation and Elaboration article discusses each checklist item and gives methodological background and published examples of transparent reporting. The STROBE checklist is best used in conjunction with this article (freely available on the Web sites of PLoS Medicine at http://www.plosmedicine.org/, Annals of Internal Medicine at http://www.annals.org/, and Epidemiology at http://www.epidem.com/). Information on the STROBE Initiative is available at www.strobe-statement.org.
